# Efficacy and safety of sequential immunotherapy following concurrent radiotherapy with S-1 in older patients with esophageal squamous cell carcinoma: an inverse probability weighting analysis

**DOI:** 10.3389/fimmu.2026.1721745

**Published:** 2026-03-11

**Authors:** Ning Yang, Jiaqi Yu, Dongyu Lei, Zimeng Zhang, Chaomang Zhu, Shixiang Zhou, Die Jiang, Hongmei Yin, Duojie Li

**Affiliations:** 1 Department of Radiotherapy, The First Affiliated Hospital of Bengbu Medical University, Bengbu, Anhui, China; 2 Anhui Provincial Key Laboratory of Tumor Evolution and Intelligent Diagnosis and Treatment, Bengbu Medical University, Bengbu, Anhui, China; 3 Joint Research Center for Regional Diseases of IHM, Bengbu Medical University, Bengbu, Anhui, China

**Keywords:** chemoradiotherapy, esophageal squamous cell carcinoma, immune checkpoint inhibitors, older patients, safety, survival

## Abstract

**Background:**

To evaluate the efficacy and safety of sequential immune checkpoint inhibitors (ICIs) following concurrent chemoradiotherapy (CCRT) in older (≥70 years) patients with locally advanced esophageal squamous cell carcinoma (ESCC).

**Methods:**

A total of 193 older patients (≥70 years) with locally advanced ESCC treated between January 2022 and December 2023 were retrospectively analyzed and divided into two groups: the CCRT group (radiotherapy with concurrent S-1, n=108) and the CCRT+ICIs group (sequential ICIs after CCRT, n=85). Baseline imbalances were adjusted via inverse probability of treatment weighting (IPTW). The primary endpoints were overall survival (OS) and progression-free survival (PFS), whereas the secondary endpoints included safety and prognostic factors.

**Results:**

After IPTW adjustment, OS in the CCRT+ICIs group tended to improve compared with that in the CCRT alone group (HR = 0.70, 95% CI: 0.48–1.04, p=0.071), although the difference was not statistically significant. In contrast, PFS was significantly improved in the CCRT+ICIs group (HR = 0.59, 95% CI: 0.42–0.84, p=0.003). Multivariate analysis identified age ≥75 years (OS: HR = 1.893; PFS: HR = 2.097), ECOG score =2 (OS: HR = 3.310; PFS: HR = 2.188), T4 stage (OS: HR = 2.221; PFS: HR = 2.080), and N3 stage (OS: HR = 3.841; PFS: HR = 2.920) as independent adverse prognostic factors. Immunotherapy-specific toxicities, including hypothyroidism and pneumonitis, occurred more frequently in the CCRT+ICIs group, which is consistent with the toxicity profile of ICIs, whereas neutropenia and vomiting were less common. A lower incidence of vomiting was also observed in the CCRT+ICIs group, although the difference did not reach statistical significance (p=0.053).

**Conclusion:**

Sequential ICI therapy after CCRT significantly improved PFS and reduced mortality risk in older ESCC patients, though close monitoring is warranted for pneumonitis and skin-related toxicities. The reduced incidence of neutropenia suggests a hematologic safety advantage of the sequential strategy. Patients aged ≥75 years, with an Eastern Cooperative Oncology Group (ECOG) score of 2, or with N3 disease constitute high-risk subgroups and warrant individualized treatment approaches.

## Introduction

Esophageal cancer ranks as the seventh most common malignancy worldwide and the sixth leading cause of cancer-related mortality (1). China accounts for more than 50% of the global burden of esophageal cancer (2), with approximately 90% of cases classified as esophageal squamous cell carcinoma (ESCC) (1). Data from 2020 indicate that China reported approximately 320,000 new cases and 300,000 deaths from esophageal cancer, with the majority occurring in older patients (2). Because early symptoms are often insidious, most patients are diagnosed at a locally advanced stage, liminating the opportunity for surgical resection (3).

For unresectable locally advanced ESCC, definitive concurrent chemoradiotherapy (CCRT) remains the standard treatment approach. In older patients, the oral fluoropyrimidine agent S-1 combined with radiotherapy has emerged as the preferred alternative to platinum-based regimens, owing to its favorable tolerability and survival benefit, with reported 3-year overall survival (OS) rates ranging from 26.9% to 55.4% (4, 5). However, nearly 50% of patients still experience disease progression, and the 5-year survival rate remains below 20% (6–8), underscoring the urgent need for more effective therapeutic strategies.

In recent years, immune checkpoint inhibitors (ICIs) have revolutionized the therapeutic landscape of esophageal cancer. They have demonstrated significant survival benefits as second-line therapy in metastatic esophageal cancer (9) and have also shown superiority when combined with chemotherapy as first-line treatment in advanced disease (10–16). Preclinical studies suggest that ICIs may act synergistically with CCRT: RTcan promote tumor antigen release and activate the cGAS-STING pathway (17), while ICIs can reverse T-cell exhaustion (18), thereby enhancing systemic antitumor immune responses.

Nevertheless, important gaps in evidence remain. First, the relative merits of concurrent versus sequential immunotherapy remain unclear; second, older patients are underrepresented in pivotal phase III clinical trials; and third, the unique genomic characteristics of Asian ESCC patients may influence immunotherapy efficacy (19). Therefore, this study aimed to apply inverse probability of treatment weighting (IPTW) to adjust for selection bias and systematically evaluate the efficacy and safety of sequential ICIs following CCRT compared with CCRT alone in patients aged ≥70 years with locally advanced ESCC.

This study aimed to correct for selection bias using inverse probability of treatment weighting (IPTW) and systematically evaluated the efficacy and safety of sequential ICIs following CCRT compared with CCRT alone in patients aged ≥70 years with locally advanced ESCC.

## Materials and methods

### Patients

A total of 221 older patients with locally advanced esophageal squamous cell carcinoma (ESCC) treated at Bengbu Medical University between January 2022 and December 2023 were enrolled in this study. For this study, ‘locally advanced’ disease was defined according to the American Joint Committee on Cancer (AJCC, 8th edition) staging system as clinical stage II (T2-T3N0M0) to stage III (T1-T4N1-3M0, or T4N0M0), deemed unsuitable for surgical resection or refusing surgery. Patients with T1N0 disease (stage I) or distant metastasis (M1, stage IV) were excluded. Eligible patients were aged ≥70 years, histologically confirmed with ESCC, had no prior systemic therapy, adequate organ function, the ability to complete follow-up, and had provided written informed consent. Patients were excluded if they had non-squamous histology, pleural metastasis, malignant effusion, high risk of gastrointestinal bleeding, esophageal fistula or perforation, malnutrition (PG-SGA ≥9), prior esophageal surgery or chemoradiotherapy, a second primary malignancy or a history of malignancy within 5 years, autoimmune disease, active infection or tuberculosis, immunodeficiency or organ transplantation, major surgery or trauma within 4 weeks before immunotherapy, or severe allergy to immune agents.

### Treatment

In the control group, patients received definitive concurrent chemoradiotherapy (CCRT), which consisted of intensity-modulated radiotherapy (6 MV IMRT, 1.8–2.0 Gy per fraction, 5 fractions/week, total dose 59.4–60.0 Gy) combined with oral S-1 (60 mg/m² per day, twice daily for 14 days followed by 7 days off, four cycles in total). In the observation group, immune checkpoint inhibitors (ICIs), including tislelizumab, camrelizumab, toripalimab, and sintilimab (200 mg intravenously every 3 weeks) or durvalumab (1500 mg intravenously every 4 weeks), all of which are PD-1/PD-L1 inhibitors, were administered sequentially after CCRT.

### Assessments

For follow-up and efficacy evaluation, laboratory tests were performed weekly during CCRT and every 3 weeks thereafter. During immunotherapy, comprehensive assessments, including physical examination, Eastern Cooperative Oncology Group (ECOG) performance status, laboratory tests, safety evaluation, and imaging assessments (contrast-enhanced chest CT, neck/abdominal ultrasound, and esophagography), were conducted every three cycles. Brain MRI, bone scan, or PET-CT was optional, and biopsy was performed if recurrence was suspected. After radiotherapy, the tumor response was assessed independently by two experienced oncologists according to RECIST 1.1. Treatment failure was classified as locoregional recurrence (esophageal or regional lymph nodes) or distant metastasis (nonregional lymph nodes or systemic spread) on the basis of histological, cytological, or unequivocal radiological evidence. Deaths were confirmed by official records.

### Efficacy evaluation and follow - up

Follow-up was completed in May 2025. Overall survival (OS) was defined as the time from treatment initiation to death or last follow-up, while progression-free survival (PFS) was defined as the time from treatment initiation to documented progression or death. Survival analyses were performed using the Kaplan–Meier method with group comparisons by the log-rank test. Independent prognostic factors were assessed by multivariate Cox regression analysis, with results reported as hazard ratios (HRs) and 95% confidence intervals (CIs). Statistical analyses were performed using SPSS version 26.0 and R version 4.2.1, and two-sided p-values <0.05 were considered statistically significant.

### Statistical analysis

To adjust for potential baseline confounding, inverse probability of treatment weighting (IPTW) was performed using the ipw package in R version 4.2.1. The propensity score was estimated from a logistic regression model that included the following pre-specified covariates: Age, Eastern Cooperative Oncology Group (ECOG) performance status, Tumor site, Tumor length (dichotomized at the cohort median of 5.55 cm), Body mass index (BMI), T stage, and N stage. Stabilized inverse probability weights were calculated and applied. The multivariate Cox proportional hazards models for overall survival (OS) and progression-free survival (PFS) were pre-specified to include the following clinically relevant variables: age, sex, Eastern Cooperative Oncology Group (ECOG) performance status, tumor site, tumor length, body mass index (BMI), T stage, and N stage. This ‘full model’ approach was adopted because all these variables are established or potential prognostic factors in esophageal cancer, and the sample size was deemed sufficient to support the stable estimation of the model with this number of covariates. The balance of baseline characteristics between the two treatment groups after weighting was assessed using absolute standardized mean differences (SMDs), with an SMD of <0.10 considered indicative of good balance. Survival analyses were performed using weighted Kaplan-Meier estimates and Cox proportional hazards models. Statistical significance was set at a two-sided p-value <0.05.

## Results

### Patient characteristics

**Figure 1:**
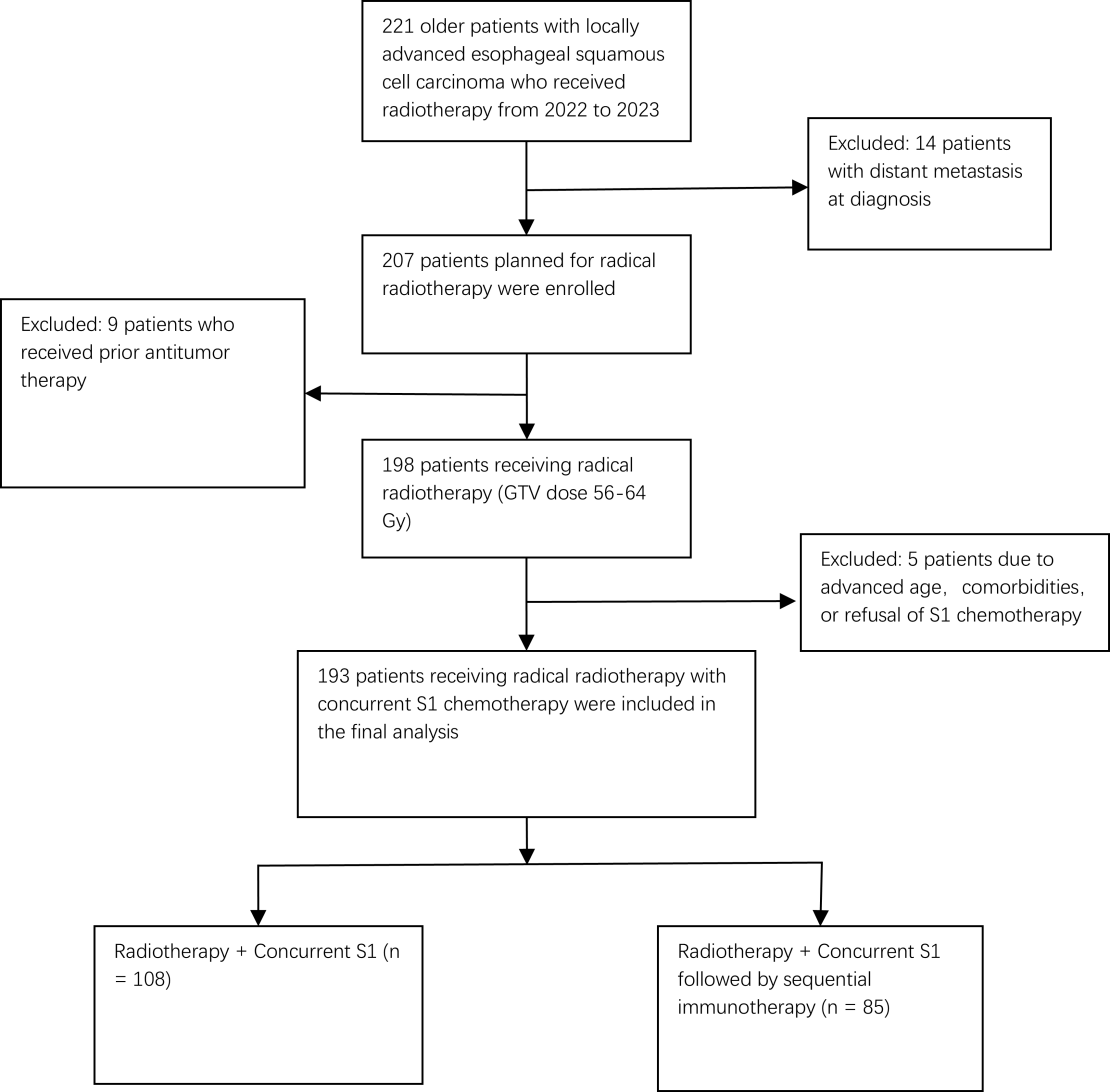
Flowchart of patient selection.

As shown in **Figure 1**, our initial cohort consisted of 221 patients who received definitive CCRT as their first-line treatment. After applying the eligibility criteria, 193 patients were included: 108 in the CCRT-only group and 85 in the CCRT+ICIs group. The baseline characteristics of the patients are summarized in **Table 1**. This retrospective study enrolled 193 older patients (aged >70 years) with locally advanced esophageal squamous cell carcinoma (ESCC), all of whom underwent S-1-based concurrent chemoradiotherapy (CCRT). Patients were categorized into two treatment cohorts: the CCRT group (S-1 chemotherapy with concurrent radiotherapy alone, n = 108) and the CCRT+ICIs group (S-1 chemotherapy with concurrent radiotherapy followed by sequential immune checkpoint inhibitor therapy, n = 85).

**Table 1:** Baseline characteristics before and after IPTW.

Characteristics	Before IPTW				After IPTW			
	Total	CCRT	CCRT+ICIs	P	Total	CCRT	CCRT+ICIs	P
Age, n (%)				0.916				0.894
70-75	90 (46.6)	50 (46.3)	40 (47.1)		90 (47)	51 (47)	39 (46)	
>75	103 (53.4)	58 (53.7)	45 (52.9)		103 (53)	57 (53)	46 (54)	
Sex, n (%)				0.328				0.592
female	81 (42.0)	42 (38.9)	39 (45.9)		82 (43)	44 (41)	38 (45)	
male	112 (58.0)	66 (61.1)	46 (54.1)		111 (57)	64 (59)	47 (55)	
ECOG, n (%)				0.061				0.994
0	60 (31.1)	28 (25.9)	32 (37.6)		60 (31)	33 (31)	26 (31)	
1	87 (45.1)	48 (44.4)	39 (45.9)		86 (45)	49 (45)	38 (44)	
2	46 (23.8)	32 (29.6)	14 (16.5)		47 (24)	26 (24)	21 (25)	
Smoking history, n (%)				0.324				0.412
no	121 (62.7)	71 (65.7)	50 (58.8)		123 (64)	72 (67)	51 (61)	
yes	72 (37.3)	37 (34.3)	35 (41.2)		69 (36)	36 (33)	33 (39)	
Alcohol history, n (%)				0.909				0.470
no	128 (66.3)	72 (66.7)	56 (65.9)		125 (65)	73 (67)	52 (62)	
yes	65 (33.7)	36 (33.3)	29 (34.1)		67 (35)	35 (33)	32 (38)	
Anemia, n (%)				0.746				0.655
no	143 (74.1)	81 (75.0)	62 (72.9)		144 (75)	82 (76)	62 (73)	
grade I	50 (25.9)	27 (25.0)	23 (27.1)		49 (25)	26 (24)	23 (27)	
Site, n (%)				0.655				0.991
upper	60 (31.1)	36 (33.3)	24 (28.2)		60 (31)	34 (31)	26 (31)	
middle	73 (37.8)	41 (38.0)	32 (37.6)		75 (39)	41 (38)	33 (39)	
distal	60 (31.1)	31 (28.7)	29 (34.1)		58 (30)	33 (30)	25 (30)	
Length (cm), n (%)				0.003				0.930
≤5.55	128 (66.3)	62 (57.4)	66 (77.6)		129 (67)	72 (66)	57 (67)	
>5.55	65 (33.7)	46 (42.6)	19 (22.4)		64 (33)	36 (34)	28 (33)	
BMI(kg/m2), n (%)				0.919				0.970
<18.5	46 (23.8)	26 (24.1)	20 (23.5)		47 (25)	26 (24)	21 (25)	
18.5-24.9	120 (62.2)	66 (61.1)	54 (63.5)		117 (61)	67 (62)	51 (60)	
>24.9	27 (14.0)	16 (14.8)	11 (12.9)		28 (14)	15 (14)	13 (15)	
T_stage, n (%)				0.774				0.944
2	52 (26.9)	27 (25.0)	25 (29.4)		47 (24)	27 (25)	20 (23)	
3	86 (44.6)	50 (46.3)	36 (42.4)		89 (46)	50 (46)	39 (46)	
4	55 (28.5)	31 (28.7)	24 (28.2)		57 (30)	31 (29)	26 (31)	
N_stage, n (%)				0.239				>0.999
0	38 (19.7)	16 (14.8)	22 (25.9)		38 (20)	21 (20)	17 (20)	
1	39 (20.2)	23 (21.3)	16 (18.8)		39 (20)	22 (20)	17 (21)	
2	82 (42.5)	47 (43.5)	35 (41.2)		82 (43)	46 (43)	36 (42)	
3	34 (17.6)	22 (20.4)	12 (14.1)		32 (17)	18 (17)	14 (17)	

CCRT, concurrent chemoradiotherapy; ICI, immune checkpoint inhibitor; ECOG, Eastern Cooperative Oncology Group; Hgb, hemoglobin; MBI, body mass index.

Before adjustment, a significant imbalance was observed between the two cohorts in terms of tumor length (p = 0.003), with the CCRT group presenting a higher proportion of longer lesions (>5.5 cm) compared with the CCRT+ICIs group (42.6% vs. 22.4%). No statistically significant differences were found in the remaining baseline characteristics (all p > 0.05), as shown in **Table 1** (baseline characteristics before and after inverse probability weighting). To minimize potential confounding bias due to such imbalances, particularly tumor length, and to enhance comparability between the groups, inverse probability of treatment weighting (IPTW) based on propensity scores was applied. Following IPTW adjustment, all assessed baseline characteristics were well balanced between the two groups, with all variables showing p-values >0.05. The effectiveness of IPTW was confirmed by assessing the balance of covariates using absolute standardized mean differences (SMDs). After weighting, the SMD for every pre-specified covariate was reduced to below the 0.10 threshold (**Supplementary Figure 1**), indicating excellent balance between the CCRT and CCRT+ICIs groups. This confirmed the effectiveness of IPTW in generating balanced analytical cohorts for subsequent efficacy and safety analyses.

### Follow-up duration

The median follow-up time for the entire cohort, estimated using the reverse Kaplan-Meier method, was 33.0 months (95% CI, 30.0 to 35.0). The median follow-up was 33.0 months (95% CI, 31.0 to 38.0) in the CCRT group and 31.0 months (95% CI, 30.0 to 35.0) in the CCRT+ICIs group. The median (interquartile range [IQR]) of the observed follow-up time was 20.0 months (IQR, 13.0–29.0) for the entire cohort, 17.0 months (IQR, 11.0–25.25) for the CCRT group, and 23.0 months (IQR, 17.0–30.0) for the CCRT+ICIs group.

### Overall survival

**Figure 2:**
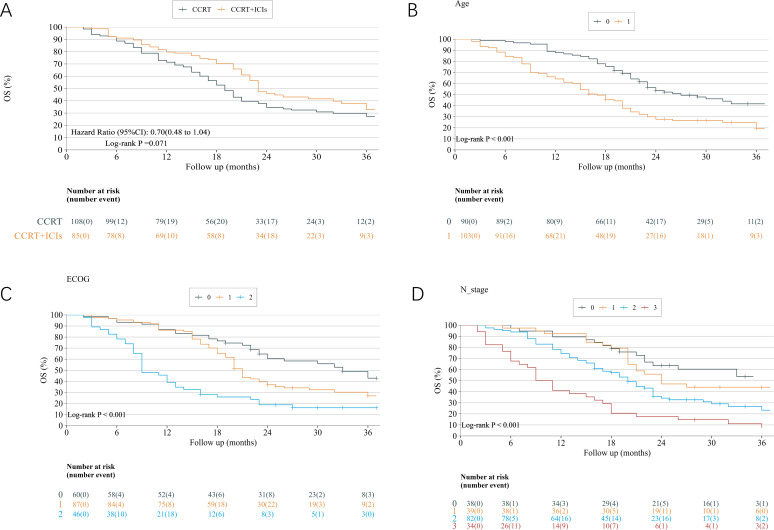
**(A)** IPTW-adjusted Kaplan–Meier OS curves for patients receiving concurrent chemoradiotherapy (CCRT) alone and CCRT followed by immune checkpoint inhibitors (CCRT+ICIs). **(B)** OS curves stratified by age: 70–75 years (curve 0) and >75 years (curve 1). **(C)** OS curves stratified by Eastern Cooperative Oncology Group (ECOG) performance status (0, 1, and 2). **(D)** OS curves stratified by N stage (N0–N3). Hazard ratios (HRs) and p-values were derived from log-rank tests. The numbers at risk are shown below each panel.

After inverse probability of treatment weighting (IPTW) adjustment, the CCRT+ICIs group demonstrated a trend toward improved overall survival (OS) compared with the CCRT alone group (HR = 0.70, 95% CI: 0.48–1.04, p = 0.071), although the difference did not reach statistical significance (**Figure 2A**). Multivariate analysis indicated that patients receiving CCRT+ICIs had a significant improvement in OS compared with those in the CCRT-only group, with a 49% reduction in the risk of death (HR = 0.511; 95% CI: 0.342–0.761; p = 0.001). Moreover, age >75 years, Eastern Cooperative Oncology Group (ECOG) performance status of 2, T4 stage, and N3 stage were identified as independent adverse prognostic factors for OS (**Table 2**). K–-M survival analysis revealed that advanced age, poor Eastern Cooperative Oncology Group (ECOG) performance status, and high N stage were significantly associated with poor OS (**Figures 2B–D**).

**Table 2:** Results of multivariate regression analysis of OS.

Variable	Estimate	Std.error	Statistic	HR (95%CI)	P
Group					
CCRT	0.000			reference	
CCRT+ICIs	-0.672	0.204	-3.298	0.511 (0.342, 0.761)	0.001
Age					
70-75	0.000			reference	
>75	0.638	0.228	2.793	1.893 (1.210, 2.962)	0.005
ECOG					
0	0.000			reference	
1	-0.105	0.272	-0.385	0.901 (0.529, 1.534)	0.700
2	1.197	0.283	4.227	3.310 (1.900, 5.767)	<0.001
Site					
upper	0.000			reference	
middle	0.326	0.367	0.890	1.386 (0.675, 2.843)	0.374
distal	0.592	0.361	1.638	1.808 (0.890, 3.671)	0.101
Length (cm)					
≤5.55	0.000			reference	
>5.55	0.232	0.248	0.935	1.261 (0.775, 2.051)	0.350
T_stage					
2	0.000			reference	
3	0.433	0.265	1.637	1.542 (0.918, 2.590)	0.102
4	0.798	0.322	2.480	2.221 (1.182, 4.173)	0.013
N_stage					
0	0.000			reference	
1	-0.231	0.388	-0.595	0.794 (0.371, 1.698)	0.552
2	0.567	0.336	1.687	1.764 (0.912, 3.409)	0.092
3	1.346	0.419	3.209	3.841 (1.689, 8.737)	0.001

CCRT, concurrent chemoradiotherapy; ICI, immune checkpoint inhibitor; ECOG, Eastern Cooperative Oncology Group.

### Progression-free survival

**Figure 3:**
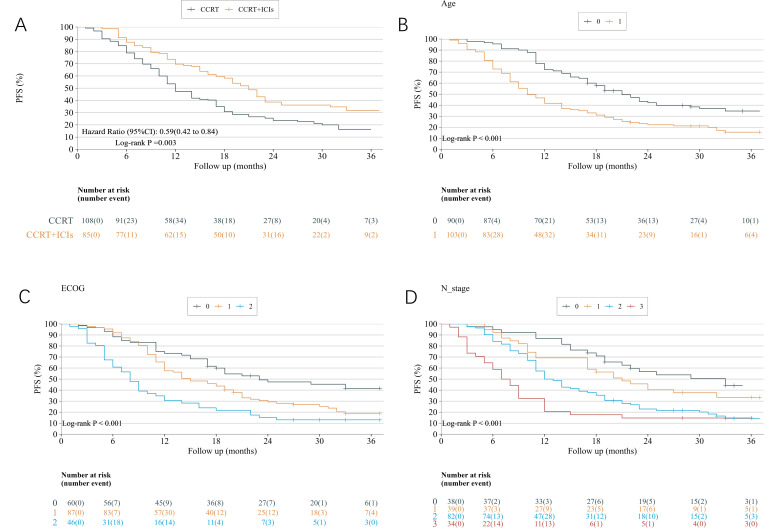
**(A)** IPTW-adjusted Kaplan–Meier PFS curves for patients receiving concurrent chemoradiotherapy (CCRT) alone and CCRT followed by immune checkpoint inhibitors (CCRT+ICIs). **(B)** PFS curves stratified by age: 70–75 years (curve 0) and >75 years (curve 1). **(C)** PFS curves stratified by Eastern Cooperative Oncology Group (ECOG) performance status (0, 1, and 2). **(D)** PFS curves stratified by N stage (N0–N3). Hazard ratios (HRs) and p-values were derived from log-rank tests. The numbers at risk are shown below each panel.

In terms of progression-free survival (PFS), the CCRT+ICIs group demonstrated a significant survival advantage over the CCRT group did (HR = 0.59, 95% CI: 0.42–0.84, p = 0.003) (**Figure 3A**). Multivariate Cox analysis further confirmed that patients in the CCRT+ICIs group exhibited a significant improvement in PFS compared with those in the CCRT-only group. The risk of disease progression or death was 56% lower in the CCRT+ICIs group than in the CCRT group (HR = 0.442; 95% CI: 0.306–0.638; p < 0.001). Age, an Eastern Cooperative Oncology Group (ECOG) score of 2, and T4, N2, and N3 stages were found to be independent prognostic factors for PFS (all p < 0.05) (**Table 3**). Kaplan–Meier survival analysis revealed that patients aged >75 years, those with an Eastern Cooperative Oncology Group (ECOG) score of 2, and those with N2 or N3 stage disease had significantly shorter PFS (**Figures 3B–D**).

**Table 3:** Results of multivariate regression analysis of PFS.

Variable	Estimate	Std.error	Statistic	HR (95%CI)	P
Group					
CCRT	0.000			reference	
CCRT+ICIs	-0.816	0.187	-4.356	0.442 (0.306, 0.638)	<0.001
Age					
70-75	0.000			reference	
> 75	0.740	0.216	3.425	2.097 (1.373, 3.203)	0.001
ECOG					
0	0.000			reference	
1	-0.143	0.259	-0.552	0.867 (0.522, 1.439)	0.581
2	0.783	0.267	2.932	2.188 (1.297, 3.694)	0.003
Site					
upper	0.000			reference	
middle	0.237	0.333	0.711	1.268 (0.659, 2.436)	0.477
distal	0.541	0.325	1.667	1.718 (0.909, 3.246)	0.095
Length (cm)					
≤5.55	0.000			reference	
>5.55	0.040	0.227	0.178	1.041 (0.668, 1.624)	0.858
T_stage					
2	0.000			reference	
3	0.276	0.235	1.171	1.317 (0.830, 2.090)	0.242
4	0.732	0.296	2.479	2.080 (1.166, 3.713)	0.013
N_stage					
0	0.000			reference	
1	-0.128	0.348	-0.367	0.880 (0.445, 1.740)	0.713
2	0.633	0.305	2.074	1.884 (1.035, 3.427)	0.038
3	1.072	0.387	2.771	2.920 (1.368, 6.231)	0.006

CCRT, concurrent chemoradiotherapy; ICI, immune checkpoint inhibitor; ECOG, Eastern Cooperative Oncology Group.

### Adverse events

Details of adverse events are presented in **Table 4**. Immune-related adverse events such as hypothyroidism, dermatitis, and pneumonitis were more frequent in the CCRT+ICIs group compared with the CCRT group, consistent with the known toxicity profile of immune checkpoint inhibitors. Conversely, neutropenia was significantly less common (p < 0.001). A lower incidence of vomiting was also observed in the CCRT+ICIs group, although the difference did not reach statistical significance (p = 0.053).

**Table 4:** Adverse reactions.

Variables	Total (n = 193)	CCRT (n = 108)	CCRT+ICIs (n = 85)	P
Leukopenia, n (%)				0.394
no	40 (20.7)	20 (18.5)	20 (23.5)	
yes	153 (79.3)	88 (81.5)	65 (76.5)	
Neutropenia, n (%)				< 0.001
no	45 (23.3)	15 (13.9)	30 (35.3)	
yes	148 (76.7)	93 (86.1)	55 (64.7)	
Anemia, n (%)				0.971
no	32 (16.6)	18 (16.7)	14 (16.5)	
yes	161 (83.4)	90 (83.3)	71 (83.5)	
Thrombocytopenia, n (%)				0.080
no	135 (69.9)	70 (64.8)	65 (76.5)	
yes	58 (30.1)	38 (35.2)	20 (23.5)	
Hypothyroidism, n (%)				0.013
no	180 (93.3)	105 (97.2)	75 (88.2)	
yes	13 (6.7)	3 (2.8)	10 (11.8)	
Nausea, n (%)				0.053
no	117 (60.6)	72 (66.7)	45 (52.9)	
yes	76 (39.4)	36 (33.3)	40 (47.1)	
Vomiting, n (%)				0.053
no	164 (85)	87 (80.6)	77 (90.6)	
yes	29 (15)	21 (19.4)	8 (9.4)	
Diarrhea, n (%)				0.414
no	161 (83.4)	88 (81.5)	73 (85.9)	
yes	32 (16.6)	20 (18.5)	12 (14.1)	
Dermatitis, n (%)				0.244
no	186 (96.4)	106 (98.1)	80 (94.1)	
yes	7 (3.6)	2 (1.9)	5 (5.9)	
Fistula, n (%)				0.696
no	187 (96.9)	104 (96.3)	83 (97.6)	
yes	6 (3.1)	4 (3.7)	2 (2.4)	
Pneumonitis, n (%)				0.008
no	145 (75.1)	89 (82.4)	56 (65.9)	
yes	48 (24.9)	19 (17.6)	29 (34.1)	

CCRT, concurrent chemoradiotherapy; ICI, immune checkpoint inhibitor.

Overall, this study suggests that sequential immunotherapy following CCRT significantly improves PFS and may provide a potential OS benefit in older patients with locally advanced ESCC, although baseline clinical characteristics such as age, performance status, and nodal burden remain critical determinants of survival outcomes.

## Discussion

This study revealed that in patients aged ≥70 years with locally advanced esophageal squamous cell carcinoma (ESCC), the sequential administration of immune checkpoint inhibitors (ICIs) following chemoradiotherapy (CCRT) significantly improved progression-free survival (PFS) and tended to improve overall survival (OS). Despite the underrepresentation of older patients in clinical trials due to comorbidities and reduced organ reserve, our findings demonstrate that this population can derive substantial benefit from sequential immunotherapy, highlighting the feasibility and potential value of ICIs in older ESCC patients.

CCRT remains the standard treatment for unresectable locally advanced esophageal cancer and has been shown to improve long-term survival in a subset of patients. S-1 is an oral fluoropyrimidine derivative composed of tegafur, gimeracil (5-chloro-2,4-dihydroxypyridine), and oteracil potassium at a molar ratio of 1:0.4:1. S-1 may also have a superior radiosensitizing effect compared to fluorouracil because the plasma half-life of fluorouracil is prolonged during S-1 treatment compared to intravenous fluorouracil, which is beneficial for radiosensitization (4).Among its components, gimeracil acts as a potent inhibitor of dihydropyrimidine dehydrogenase (DPD), thereby preventing 5-FU catabolism and enhancing radiosensitivity by suppressing DNA repair following radiation-induced damage (4). Evidence from multicenter phase II/III randomized controlled trials in older patients with esophageal squamous cell carcinoma (ESCC) has demonstrated that S-1 combined with radiotherapy significantly improves survival outcomes while maintaining a manageable toxicity profile compared with radiotherapy alone (4, 5, 20, 21). Furthermore, its oral administration provides a distinct advantage in terms of convenience over intravenous regimens, particularly for older patients and those requiring prolonged treatment. On this basis, S-1–based chemoradiotherapy has been established as a standard treatment modality for older patients with locally advanced, unresectable ESCC. Nevertheless, the risks of recurrence and distant metastasis remain substantial, and the overall 5-year survival rate continues to fall below 20% (6).

Recent evidence suggests a potential synergistic effect between radiotherapy and immunotherapy (22–24): radiotherapy not only exerts direct cytotoxic effects on local tumors but also promotes tumor antigen release and activates the cGAS-STING pathway, Reshape the tumor microenvironment (21),enhancing systemic antitumor immunity (17). Additionally, PD-1/PD-L1 blockade can reverse radiotherapy-induced T-cell exhaustion, further amplifying antitumor immune responses. Consequently, the combination or sequential use of radiotherapy and ICIs is considered a promising strategy with synergistic potential.

To our knowledge, this study is the first to specifically evaluate older patients (≥70 years) with locally advanced ESCC. Previous pivotal phase III immunotherapy trials have generally underrepresented older patients, resulting in limited evidence regarding efficacy and safety in this population (14, 25). Our results show that sequential ICIs after CCRT reduce the risk of disease progression or death by 56% (HR = 0.442, p < 0.001), with a trend toward improved OS (HR = 0.70, p = 0.071). Multivariate analysis further confirmed that ICIs consolidation independently confers an OS benefit (HR = 0.511, p = 0.001). These findings align with previous studies of immunotherapy in advanced esophageal cancer (11, 12, 15), indicating that ICIs maintain antitumor activity even in older individuals. Balancing efficacy and safety in older patients thus remains a critical consideration in clinical practice.

Placing our findings in the context of existing literature specifically focused on older patients with LA-ESCC is crucial. Prior landmark studies in this specific population, such as the multicenter randomized trial by Ji et al. (4)and the work by Wang et al. (5), have established S-1-based concurrent chemoradiotherapy as a standard of care, reporting 3-year overall survival rates of approximately 40%. Our study builds directly upon this foundation by introducing a novel therapeutic sequence: sequential immune checkpoint inhibition following the same S-1-based CCRT backbone. While the aforementioned studies defined the efficacy of the foundational regimen, our research provides the first real-world evidence addressing the clinical question of ‘what’s next’ after CCRT in this vulnerable population. The significant improvement in PFS (HR = 0.59) and the trend towards improved OS (HR = 0.70) observed in our cohort, coupled with a manageable safety profile, suggest that sequential ICIs represent a viable strategy to build upon the established standard. This positions our study as a direct evolution in the treatment paradigm for older LA-ESCC, moving from defining the optimal chemotherapy partner for radiotherapy to exploring consolidation strategies that may further extend survival.

Several independent prognostic factors were identified. Age >75 years, ECOG performance status of 2, and T4/N2–N3 stage were significantly associated with poorer OS and PFS, indicating that baseline tumor burden and functional status remain key determinants of prognosis, even in the immunotherapy era. These findings provide a rationale for individualized treatment strategies and suggest that older patients with high tumor burdens may benefit from more intensive supportive care.

Different immunotherapy combination approaches may have distinct efficacy and safety profiles. Early clinical studies indicate that concurrent CCRT combined with camrelizumab achieves higher response rates with acceptable tolerability (23, 26). However, concurrent regimens may increase the risk of acute toxicity, particularly in older patients. The results of another study indicated that the combination of immune checkpoint inhibitors (ICIs) and chemoradiotherapy (CRT) has a significant effect on survival rates. Pairwise comparisons revealed that sequential treatment with chemoradiotherapy and immune checkpoint inhibitors is more effective than concurrent treatment (27).

In this study, the CCRT+ICIs group presented increased incidences of immune-related adverse events (irAEs), including hypothyroidism, dermatitis, and pneumonitis, although most were grade 1–2 and manageable. The observed toxicity spectrum was consistent with prior large-scale immunotherapy trials and known ICI toxicity profiles (10, 28, 29). Interestingly, the incidence of neutropenia and vomiting was lower in the combination group, potentially reflecting reduced cumulative chemotherapy toxicity during the immunotherapy maintenance phase post-CCRT. Overall, sequential ICIs following CCRT demonstrated an acceptable safety profile in older patients, albeit with the need for careful monitoring of irAEs.

A noteworthy methodological finding was the difference in the statistical significance of the OS benefit between the IPTW-adjusted univariate analysis (p=0.071) and the multivariable Cox regression (p=0.001). This is not contradictory but elucidative. The IPTW-adjusted analysis confirms a strong trend favoring the CCRT+ICIs strategy at the group level. The multivariable model, by simultaneously adjusting for several strong independent prognostic factors (including age ≥75 years, ECOG status of 2, and N3 stage), provides a more precise estimate of the independent treatment effect. The significant hazard reduction (HR = 0.511, p=0.001) in this model indicates that the survival advantage associated with sequential ICIs is robust and persists after accounting for the substantial baseline mortality risk conferred by these adverse clinical features.

The findings of this study should be interpreted with caution in view of several limitations. As a single-center retrospective analysis, this study is inherently vulnerable to selection bias and unmeasured confounders. Although inverse probability of treatment weighting (IPTW) was employed to balance observable baseline characteristics, unaccounted factors—such as comorbidities, subtle differences in functional status, and individualized clinical decision-making—may still have influenced outcomes, thereby limiting the generalizability of the results. Moreover, the relatively small sample size, particularly the low proportion of patients aged over 75 years, restricts the statistical power of subgroup analyses. The scarcity of very older patients makes it difficult to draw firm conclusions regarding the efficacy and safety of sequential immunotherapy in this population. Larger, multicenter studies with broader cohorts will be required to validate these findings and to determine whether the observed benefits can be consistently reproduced across different older subgroups.

Another important issue is the heterogeneity of immune checkpoint inhibitors administered, which reflects real-world clinical practice. Differences in drug-specific efficacy and safety profiles may have influenced patient outcomes in this study. To more accurately define the role of sequential immunotherapy after chemoradiation, future prospective trials employing a uniform ICI regimen are essential. In addition, the relatively short follow-up period in this cohort restricted the evaluation of long-term survival and late-onset toxicities. Given the possibility of delayed therapeutic benefits associated with immunotherapy and the potential for late recurrence in patients with esophageal cancer, extended follow-up is needed to determine whether the observed improvement in progression-free survival can be translated into durable overall survival gains and to provide a more comprehensive understanding of the long-term safety profile.

A potential limitation is the use of a single statistical method (IPTW) for confounding control. The reviewer suggested a sensitivity analysis using propensity score matching (PSM) to test the robustness of our findings. While PSM is a valuable technique, it was not adopted here due to methodological considerations specific to our cohort. Applying PSM would necessitate excluding a substantial number of patients from the larger CCRT-alone group to match the smaller CCRT+ICIs group, leading to a significant loss of statistical power and potentially reducing the representativeness of our findings, particularly for key subgroups. Our pre-specified IPTW approach successfully balanced all baseline covariates (SMD <0.10), and the consistency of the PFS benefit across subgroups supports the robustness of our primary conclusion. Therefore, we are confident that the treatment effect we observed is unlikely to be substantially altered by the choice of analytical method.

Despite these limitations, this study provides important real-world evidence supporting the feasibility of sequential ICIs after chemoradiation in older patients with locally advanced ESCC. Validation through large-scale, multicenter, randomized controlled trials—such as ​RATIONALE 311 (30)​ and ​ESCORT-1st (12), which are specifically focused on ESCC—will be critical to define the role of consolidation immunotherapy in this vulnerable population. Furthermore, results from other pivotal trials that included substantial subgroups of older patients, such as the adjuvant ​CheckMate 577​ study (nivolumab) (25)and the definitive chemoradiation combination trial ​KEYNOTE-975​ (pembrolizumab) (31), will provide additional insights into the efficacy and safety of integrating ICIs across different treatment modalities and sequences in locally advanced ESCC.

In conclusion, sequential ICIs following CCRT significantly improve PFS and may confer OS benefit in older patients with locally advanced ESCC, with an overall manageable safety profile. These findings offer a novel approach for comprehensive treatment strategies in older individuals. Future multicenter, prospective, randomized controlled trials are warranted to validate these findings and to explore predictive biomarkers for optimizing individualized therapy.

## Data Availability

The original contributions presented in the study are included in the article/**Supplementary Material**. Further inquiries can be directed to the corresponding author.
