# Supplementary material for: Efficacy and safety of sequential immunotherapy following concurrent radiotherapy with S-1 in older patients with esophageal squamous cell carcinoma: an inverse probability weighting analysis
Source: Front Immunol. 2026 Mar 11;17:1721745. doi: 10.3389/fimmu.2026.1721745 (PMC13013532; doi:10.3389/fimmu.2026.1721745)
Supplement: Supplementary file 1 [file DataSheet1.docx]

**Supplementary Table S1： Detailed grading of adverse events.**

| **Variables** | **Total (n = 193)** | **CCRT (n = 108)** | **CCRT+ICIs (n = 85)** | **p** |
| --- | --- | --- | --- | --- |
| Leukopenia_grade, n (%) |  |  |  | 0.065 |
| 0 | 40 (20.7) | 20 (18.5) | 20 (23.5) |  |
| 1 | 104 (53.9) | 53 (49.1) | 51 (60) |  |
| 2 | 29 (15) | 23 (21.3) | 6 (7.1) |  |
| 3 | 10 (5.2) | 5 (4.6) | 5 (5.9) |  |
| 4 | 8 (4.1) | 6 (5.6) | 2 (2.4) |  |
| 5 | 2 (1) | 1 (0.9) | 1 (1.2) |  |
| Neutropenia_grade, n (%) |  |  |  | 0.013 |
| 0 | 45 (23.3) | 15 (13.9) | 30 (35.3) |  |
| 1 | 68 (35.2) | 42 (38.9) | 26 (30.6) |  |
| 2 | 48 (24.9) | 28 (25.9) | 20 (23.5) |  |
| 3 | 22 (11.4) | 15 (13.9) | 7 (8.2) |  |
| 4 | 7 (3.6) | 6 (5.6) | 1 (1.2) |  |
| 5 | 3 (1.6) | 2 (1.9) | 1 (1.2) |  |
| Anemia_grade, n (%) |  |  |  | 0.998 |
| 0 | 32 (16.6) | 18 (16.7) | 14 (16.5) |  |
| 1 | 87 (45.1) | 49 (45.4) | 38 (44.7) |  |
| 2 | 43 (22.3) | 24 (22.2) | 19 (22.4) |  |
| 3 | 19 (9.8) | 10 (9.3) | 9 (10.6) |  |
| 4 | 12 (6.2) | 7 (6.5) | 5 (5.9) |  |
| Thrombocytopenia_grade, n (%) |  |  |  | 0.486 |
| 0 | 135 (69.9) | 70 (64.8) | 65 (76.5) |  |
| 1 | 31 (16.1) | 20 (18.5) | 11 (12.9) |  |
| 2 | 13 (6.7) | 8 (7.4) | 5 (5.9) |  |
| 3 | 9 (4.7) | 6 (5.6) | 3 (3.5) |  |
| 4 | 5 (2.6) | 4 (3.7) | 1 (1.2) |  |
| Hypothyroidism_grade, n (%) |  |  |  | 0.032 |
| 0 | 180 (93.3) | 105 (97.2) | 75 (88.2) |  |
| 1 | 9 (4.7) | 2 (1.9) | 7 (8.2) |  |
| 2 | 4 (2.1) | 1 (0.9) | 3 (3.5) |  |
| Nausea_grade, n (%) |  |  |  | 0.358 |
| 0 | 117 (60.6) | 72 (66.7) | 45 (52.9) |  |
| 1 | 40 (20.7) | 19 (17.6) | 21 (24.7) |  |
| 2 | 23 (11.9) | 10 (9.3) | 13 (15.3) |  |
| 3 | 8 (4.1) | 4 (3.7) | 4 (4.7) |  |
| 4 | 5 (2.6) | 3 (2.8) | 2 (2.4) |  |
| Vomiting_grade, n (%) |  |  |  | 0.275 |
| 0 | 164 (85) | 87 (80.6) | 77 (90.6) |  |
| 1 | 20 (10.4) | 15 (13.9) | 5 (5.9) |  |
| 2 | 6 (3.1) | 4 (3.7) | 2 (2.4) |  |
| 3 | 3 (1.6) | 2 (1.9) | 1 (1.2) |  |
| Diarrhea_grade, n (%) |  |  |  | 0.784 |
| 0 | 161 (83.4) | 88 (81.5) | 73 (85.9) |  |
| 1 | 21 (10.9) | 14 (13) | 7 (8.2) |  |
| 2 | 9 (4.7) | 5 (4.6) | 4 (4.7) |  |
| 3 | 2 (1) | 1 (0.9) | 1 (1.2) |  |
| Dermatitis_grade, n (%) |  |  |  | 0.353 |
| 0 | 186 (96.4) | 106 (98.1) | 80 (94.1) |  |
| 1 | 4 (2.1) | 1 (0.9) | 3 (3.5) |  |
| 2 | 3 (1.6) | 1 (0.9) | 2 (2.4) |  |
| Fistula_grade, n (%) |  |  |  | 1.000 |
| 0 | 187 (96.9) | 104 (96.3) | 83 (97.6) |  |
| 1 | 5 (2.6) | 3 (2.8) | 2 (2.4) |  |
| 2 | 1 (0.5) | 1 (0.9) | 0 (0) |  |
| Pneumonitis_grade, n (%) |  |  |  | 0.099 |
| 0 | 145 (75.1) | 89 (82.4) | 56 (65.9) |  |
| 1 | 27 (14) | 11 (10.2) | 16 (18.8) |  |
| 2 | 7 (3.6) | 2 (1.9) | 5 (5.9) |  |
| 3 | 10 (5.2) | 4 (3.7) | 6 (7.1) |  |
| 4 | 4 (2.1) | 2 (1.9) | 2 (2.4) |  |
| CVD_grade, n (%) |  |  |  | 0.827 |
| 0 | 173 (89.6) | 97 (89.8) | 76 (89.4) |  |
| 1 | 7 (3.6) | 3 (2.8) | 4 (4.7) |  |
| 2 | 9 (4.7) | 5 (4.6) | 4 (4.7) |  |
| 3 | 4 (2.1) | 3 (2.8) | 1 (1.2) |  |
| Leukopenia_grade_3_and_higher, n (%) |  |  |  | 0.701 |
| 0 | 173 (89.6) | 96 (88.9) | 77 (90.6) |  |
| 1 | 20 (10.4) | 12 (11.1) | 8 (9.4) |  |
| Neutropenia_grade_3_and_higher, n (%) |  |  |  | 0.047 |
| 0 | 161 (83.4) | 85 (78.7) | 76 (89.4) |  |
| 1 | 32 (16.6) | 23 (21.3) | 9 (10.6) |  |
| Anemia_grade_3_and_higher, n (%) |  |  |  | 0.891 |
| 0 | 162 (83.9) | 91 (84.3) | 71 (83.5) |  |
| 1 | 31 (16.1) | 17 (15.7) | 14 (16.5) |  |
| Thrombocytopenia_grade_3_and_higher, n (%) |  |  |  | 0.226 |
| 0 | 179 (92.7) | 98 (90.7) | 81 (95.3) |  |
| 1 | 14 (7.3) | 10 (9.3) | 4 (4.7) |  |
| Hypothyroidism_grade_3_and_higher, n (%) |  |  |  | 1.000 |
| 0 | 193 (100) | 108 (100) | 85 (100) |  |
| Nausea_grade_3_and_higher, n (%) |  |  |  | 0.874 |
| 0 | 180 (93.3) | 101 (93.5) | 79 (92.9) |  |
| 1 | 13 (6.7) | 7 (6.5) | 6 (7.1) |  |
| Vomiting_grade_3_and_higher, n (%) |  |  |  | 1.000 |
| 0 | 190 (98.4) | 106 (98.1) | 84 (98.8) |  |
| 1 | 3 (1.6) | 2 (1.9) | 1 (1.2) |  |
| Diarrhea_grade_3_and_higher, n (%) |  |  |  | 1.000 |
| 0 | 191 (99) | 107 (99.1) | 84 (98.8) |  |
| 1 | 2 (1) | 1 (0.9) | 1 (1.2) |  |
| Dermatitis_grade_3_and_higher, n (%) |  |  |  | 1.000 |
| 0 | 193 (100) | 108 (100) | 85 (100) |  |
| Fistula_grade_3_and_higher, n (%) |  |  |  | 1.000 |
| 0 | 193 (100) | 108 (100) | 85 (100) |  |
| Pneumonitis_grade_3_and_higher, n (%) |  |  |  | 0.305 |
| 0 | 179 (92.7) | 102 (94.4) | 77 (90.6) |  |
| 1 | 14 (7.3) | 6 (5.6) | 8 (9.4) |  |
| CVD_grade_3_and_higher, n (%) |  |  |  | 0.632 |
| 0 | 189 (97.9) | 105 (97.2) | 84 (98.8) |  |
| 1 | 4 (2.1) | 3 (2.8) | 1 (1.2) |  |

Incidence of adverse events graded according to Common Terminology Criteria for Adverse Events (CTCAE) version 5.0. Data are presented as number of patients (n) and percentage (%) for each grade (0-5) within each treatment group. The p-value is for the comparison of the overall distribution of adverse event grades between the two groups. CCRT, concurrent chemoradiotherapy; ICIs, immune checkpoint inhibitors.

**Supplementary Figure S1. Assessment of Covariate Balance Before and After Inverse Probability of Treatment Weighting (IPTW) Using Standardized Mean Differences (SMDs).**

The plot displays the absolute SMD for each baseline variable. The red line with circles represents the ‘Unmatched’ cohort, and the green line with triangles represents the cohort after IPTW adjustment. The vertical dashed line indicates the commonly accepted imbalance threshold of SMD = 0.2. After IPTW, the SMDs for all covariates fell below 0.1, indicating excellent balance was achieved.
